# FACTORS ASSOCIATED WITH VOLUNTEERING AMONG FAMILY CAREGIVERS OF OLDER ADULTS

**DOI:** 10.1093/geroni/igac059.3119

**Published:** 2022-12-20

**Authors:** Sol Baik, Rachel Coleman, Jennifer Crittenden

**Affiliations:** University of Virginia Weldon Cooper Center for Public Service, Charlottesville, Virginia, United States; University of Maine, Bangor, Maine, United States; University of Maine, Bangor, Maine, United States

## Abstract

Family caregiving has been linked to an increased risk of poor mental health, poor physical health, and higher rates of perceived social isolation among caregivers. Despite the connection between caregiving and negative outcomes, or perhaps because of this connection, caregivers seek out and enjoy other life roles and activities including formal volunteerism. To explore the connection between informal caregiving and volunteering and establish the representative prevalence of formal volunteering among caregivers, descriptive and multivariate logistic regression analyses were carried out with data from 1,745 caregivers in the National Study of Caregiving (NSOC) (2017). Utilizing social capital theory four models were constructed with salient demographic characteristics associated with volunteering (model 1), caregiving context (model 2), caregiver physical and mental health (model 3), along with participation in informal and formal social networks (model 4). About a quarter of the sample participated in volunteering (26%). The average age was 60.5 years (SD = 14.3) and more than half consisted of female caregivers (67.11%). Non-Hispanic Whites (62.9%) were the majority of the sample, followed by non-Hispanic Blacks (28%), Hispanic (6.5%), and caregivers in other racial/ethic groups (2.6%). Gender, educational achievement, caregiving for a spouse, coresiding with care recipient, caregiving for multiple care recipients, quality of relationship with care recipient, caregiver psychological well-being, having emotional/physical support, attending religious services, and group activity participation were all significant indicators for caregiver volunteerism. Findings support the importance of both human and social capital in volunteering among caregivers.

